# Tissue Plasminogen Activator (tPA) Use in Persistent Loculated Ascites

**DOI:** 10.7759/cureus.72331

**Published:** 2024-10-24

**Authors:** Komail Mujtaba Ali, Rhett Molloy, Alexander Friedman, Wichit Srikureja, Christopher Bent, Roger C Garrison

**Affiliations:** 1 Internal Medicine, Riverside University Health System Medical Center, Moreno Valley, USA; 2 Gastroenterology and Hepatology, Riverside University Health System Medical Center, Moreno Valley, USA; 3 Diagnostic Radiology, Riverside University Health System Medical Center, Moreno Valley, USA

**Keywords:** decompensated liver cirrhosis, loculated ascites, refractory ascites, tissue plasminogen activator (tpa), tpa

## Abstract

Spontaneous bacterial peritonitis (SBP) complicated by loculated ascites presents a therapeutic challenge, particularly when standard of care or surgical intervention is not feasible. This case report documents the successful use of intraperitoneal tissue plasminogen activator (tPA) as adjunctive salvage therapy in an adult female with decompensated liver cirrhosis and loculated infected ascites. After no improvement in the patient’s clinical condition following 14 days of intravenous antibiotics, catheter-directed intraperitoneal tPA was administered for three days, resulting in the improvement of her abdominal pain and resolution of the loculations. This case provides additional support for the potential efficacy of tPA as salvage therapy in managing loculated infected ascites in cirrhotic patients who have failed standard treatments.

## Introduction

Ascites is a common complication of cirrhosis. More than half of patients with compensated cirrhosis will develop ascites within 10 years. The development of ascites portends a poor prognosis with a two-year mortality rate of up to 40% [1]. Spontaneous bacterial peritonitis (SBP) is a severe complication of ascites, usually occurring in patients with advanced cirrhosis, with prevalence rates of 10-30% in hospitalized patients [2,3]. Early treatment is paramount to ensure a good outcome. Delays in diagnosis and treatment lead to a 2-3-fold increased risk of death. Despite adequate therapy with antibiotics, the mortality rate ranges from 10-46% [2,4]. Cirrhotic patients may develop loculated ascites. While uncommon, loculated ascites are typically encountered in settings of adhesions, peritoneal carcinomatosis, or secondary peritonitis [5]. Loculations caused by SBP are rarely reported and may reflect delayed detection and treatment of the infection. The etiology and mechanism of development remain poorly understood. It is hypothesized that loculations develop due to increased fibrin deposition with the increased release of fibrogenic cytokines [6]. Paracentesis in patients with loculated ascites is challenging due to the fibrin strands and poor communication between the loculated pockets. Fibrin strands in loculated ascites markedly impair antibiotic diffusion [7], which may delay adequate therapy and further increase the risks of complications and mortality. Adequate thrombolysis of the fibrin strands can potentially improve treatment outcomes of SBP in cirrhotic patients with loculated ascites who have failed standard treatment with intravenous antibiotics.

Tissue plasminogen activator (tPA) is a serine protease that catalyzes the conversion of plasminogen to plasmin, the primary enzyme involved in the dissolution of blood clots [8] formed by the aggregation of activated platelets within fibrin meshes. Studies done in 2009 and 2011 demonstrated the efficacy of intrapleural fibrinolytic tPA in the treatment of loculated parapneumonic effusions, which has since become the standard of practice [9, 10]. While commonly used in the thoracic cavity, there have only been a few case reports concerning the use of fibrinolytic therapy for loculated fluid collections in abdominopelvic compartments. Two recent case reports demonstrate the potential use of tPA [11] and urokinase [7] as adjunctive treatments for loculated ascites in cirrhotic patients.

## Case presentation

**Figure 1:**
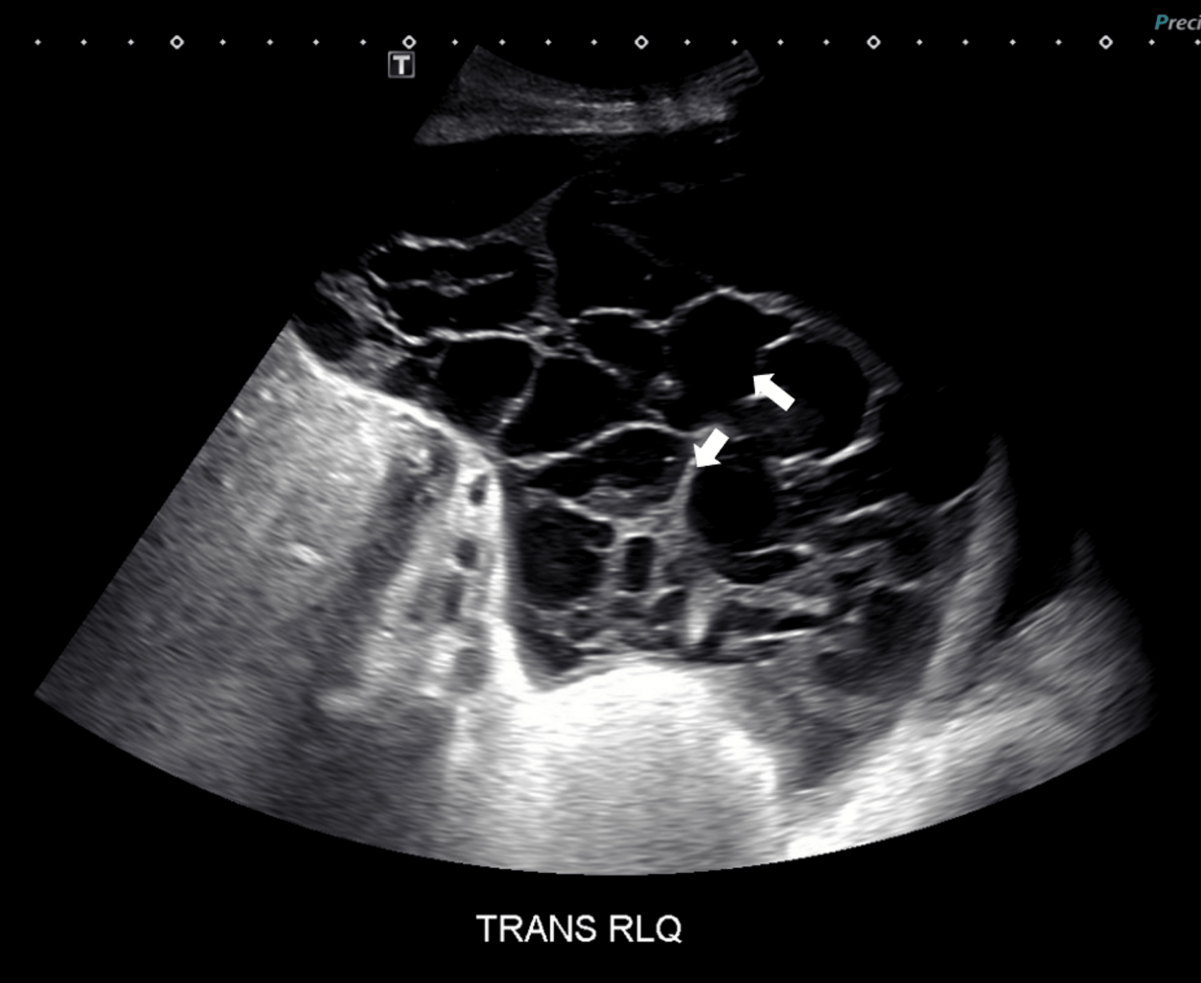
Abdominal ultrasound demonstrating loculated ascitic fluid before catheter-directed tPA administration. Arrows indicate septations between loculated ascitic fluid collections. tPA: Tissue Plasminogen Activator.

A 62-year-old female with a history of decompensated cryptogenic cirrhosis presented with confusion and abdominal pain for one week. Upon admission, her cirrhosis was classified as Child Class C with a Model for End-Stage Liver Disease (MELD) score of 34. A paracentesis performed in the ED removed 2,200 mL of hazy, yellow fluid containing 583 WBC/cumm with 367 PMN/cumm. The patient was diagnosed with SBP and treated with antibiotics for 14 days (primarily ceftriaxone and metronidazole) after an abdominal ultrasound revealed extensively loculated ascites (Figure 1). Ascitic fluid cultures were negative for bacterial, fungal, and mycobacterial organisms. On day 13, a second paracentesis by Interventional Radiology removed 500 mL of straw-colored fluid containing 300 WBC/cumm with 39 PMN/cumm. Due to the loculated nature of the patient’s ascites, there was high clinical suspicion that the fluid sample might not represent all fluid pockets. Further ascitic fluid removal was limited by the extensive loculations. On hospital day 14, with the patient’s abdominal pain and loculated fluid collections unimproved and not amenable to further conventional paracentesis, surgical intervention was considered but deemed too risky. Two peritoneal catheters with accordion drains were then inserted into the left and right lower abdominal quadrants by Interventional Radiology, and 2 mg of tPA was administered through each catheter twice a day for a total of three days. Each tPA infusion was followed by a 10-cc saline flush and locked in place for two hours before being unclamped and monitored for output. The fibrinolysis protocol utilized for this patient was adapted from prior studies on pleural effusion fibrinolysis and a case report demonstrating the use of urokinase for loculated peritoneal effusion [7, 10, 12, 13]. tPA was selected as the fibrinolytic agent due to formulary availability.

**Figure 2:**
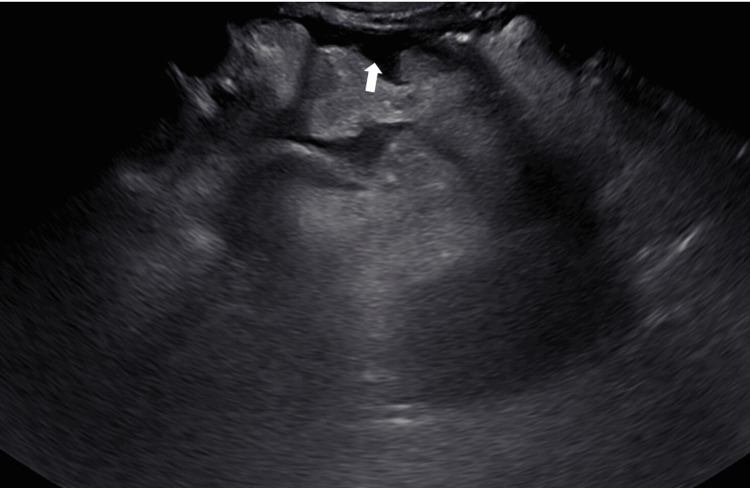
Repeat abdominal ultrasound showing resolution of loculated ascites post-tPA therapy, indicated by an arrow. tPA: Tissue Plasminogen Activator.

The two peritoneal catheters were kept in place for a total of four days. Peritoneal catheter drainage volumes are recorded in Table 1. Ceftriaxone and metronidazole were continued while the peritoneal catheters were in place. The patient reported gradual improvement in her abdominal pain, and a repeat abdominal ultrasound performed 15 days after the procedure showed resolution of the abdominopelvic loculations (Figure 2). While there were no obvious complications or signs of bleeding associated with the administration of intraperitoneal tPA, the patient’s hemoglobin did downtrend from 7.2 g/dL to 6.8 g/dL two days after completion of the three-day course of fibrinolytic therapy. The patient was transfused one unit of packed red blood cells with improvement of the hemoglobin to 7.6 g/dL, which remained stable. The patient was later discharged to a long-term acute care facility after a prolonged hospitalization complicated by her multiple other comorbid conditions.

**Table 1:** Peritoneal drain output. RLQ: Right lower quadrant; LLQ: Left lower quadrant.

Hospital Day	RLQ Peritoneal Drain Volume (mL)	LLQ Peritoneal Drain Volume (mL)
16	500	130
17	100	30
18	30	0
19	15	0

## Discussion

There are few published reports on the use of intra-abdominal fibrinolytics. Zuckerman DA et al. (2009) [9] and Rahman NM et al. (2011) [10] investigated the efficacy of tPA in treating complex parapneumonic effusions, demonstrating its effectiveness in improving patient outcomes. These findings underscore the promising role of fibrinolytic therapy, particularly with tPA, in addressing loculated effusions, laying the groundwork for similar treatments in other anatomical compartments, such as the abdominopelvic region. tPA has become a common approach for managing complex pleural effusions and occluded central venous catheters, while its use in treating symptomatic loculated peritoneal collections remains relatively uncommon.

A recent case study by Alkhero M et al. (2021) [11] demonstrated the efficacy of catheter-directed tPA in resolving complex loculations within abdominopelvic ascites. Tripon S et al. (2021) [7] demonstrated the use of intraabdominal urokinase in treating loculated ascites in cirrhosis with effective resolution of the loculations; however, the use of fibrinolytics, especially in cirrhotic patients, carries significant risks. The most concerning complication is bleeding, as these patients are already at increased risk of coagulopathy. The introduction of tPA or other fibrinolytic agents can exacerbate this risk, potentially leading to hemorrhage within the peritoneal cavity. Additionally, indwelling catheters, which are necessary for the delivery of intraperitoneal fibrinolytics, increase the risk of infection, particularly in patients with cirrhosis who are already prone to SBP. Given these risks, the use of intraperitoneal catheter-directed thrombolytic therapy should be approached with caution. Patients with loculated ascites usually have an overall poor prognosis due to underlying liver cirrhosis, necessitating a careful evaluation of the risks and benefits of this therapy.

## Conclusions

This case report adds to the literature describing the use of fibrinolytic therapy in treating loculated ascites among patients with liver cirrhosis, highlighting the importance of a multidisciplinary approach and innovative, minimally invasive techniques. In this case, the improved outcomes from catheter-directed tPA therapy suggest it could be a valuable salvage therapy for loculated infected ascites after the failure of standard treatment, particularly in patients who are suboptimal surgical candidates. In patients with loculated infected ascites where all other therapies have failed, peritoneal catheter with tPA administration may be considered as a salvage therapy, which could potentially be lifesaving. While early case reports show promise, this approach requires further evaluation through research studies to clarify its efficacy and safety profile.
